# Antimicrobial resistance profiles of *Campylobacter* spp. isolated from retail meat in Puerto Rico

**DOI:** 10.1128/mra.00821-25

**Published:** 2025-09-30

**Authors:** Gustavo Quiñones Justiniano, Esther M. Vega Quiñones, Edward G. Dudley, Nkuchia M. M'ikanatha, Yadira Malavez

**Affiliations:** 1 Department of Natural Sciences, University of Puerto Rico at Aguadilla 19847, Aguadilla, Puerto Rico; 2 Department of Food Sciences, The Pennsylvania State University 8082, University Park, Pennsylvania, USA; 3 Pennsylvania Department of Health, Harrisburg, Pennsylvania, USA; 4 Department of Biology, Biotechnology Program, University of Puerto Rico at Mayagüez, Mayagüez, Puerto Rico; Wellesley College, Wellesley, Massachusetts, USA

**Keywords:** *Campylobacter*, antimicrobial resistance, retail meat, genome analysis, phenotypic identification

## Abstract

Whole-genome sequencing and antimicrobial susceptibility testing were performed on 29 *Campylobacter* isolates from retail meat in Puerto Rico. Resistance to tetracycline, ciprofloxacin, clindamycin, and florfenicol was observed. Multidrug resistance was linked to co-occurrence of *tet(O*), *gyrA* mutations, and *cmeABC*, highlighting the public health risk of antimicrobial-resistant *Campylobacter* in retail meat.

## ANNOUNCEMENT

*Campylobacter* spp. is a leading cause of bacterial gastroenteritis globally, with rising antimicrobial resistance (AMR) complicating treatment, particularly in vulnerable populations (1, 2). In Puerto Rico, 130 cases were reported between 2022 and 2023 (3). Retail meat is a recognized source of antibiotic-resistant bacteria (4). This is the first report on *Campylobacter* prevalence and AMR profiles in chicken, turkey, and beef in Puerto Rico, based on 2 years of the National Antimicrobial Resistance Monitoring System (NARMS) (5).

A total of 306 fresh retail meat samples (chicken, *n* = 120; giblets, *n* = 6; ground beef, *n* = 48; turkey, *n* = 72) were collected monthly from supermarkets in Puerto Rico between January 2022 and December 2023. Sampling locations were based on randomized zip codes provided by the Food and Drug Administration–Center for Veterinary Medicine (FDA-CVM). *Campylobacter* isolation followed FDA-NARMS methods, including enrichment in double-strength Bolton broth, incubated under microaerophilic conditions (85% N₂, 10% CO₂, and 5% O₂) at 42°C for 24 h, streaked onto Campy-Cefex agar using cotton swabs, subcultured on blood agar, and stored at −80°C in Brucella broth with 10% glycerol (6). Phenotypic resistance was determined with SensiTitre CMV-CAMPY plates (Thermo Fisher Scientific, Waltham, MA, USA) according to EUCAST guidelines (7, 8). Genomic DNA was extracted using the DNeasy Blood Kit (Qiagen, Hilden, Germany) from 1 mL cultures grown in LB broth for 24 h at 42°C. Illumina sequencing libraries were prepared using the Nextera XT DNA Library Preparation Kit (Illumina, San Diego, CA, USA) following the manufacturer’s instructions, without modifications. Whole-genome sequencing was conducted on the MiSeq platform (Illumina, San Diego, CA, USA) with 2 × 250 bp paired-end reads. Raw reads were trimmed with Trimmomatic and assembled using SKESA via MicroRunQC v1.2 (GalaxyTrakr) (9). AMR genes were identified with Staramr v0.11.0. Default parameters were used except where otherwise noted. Species identification and sequence typing were performed using the NCBI Pathogen Detection platform.

Forty-nine *Campylobacter*-positive samples were recovered; 29 were selected for WGS. *C. coli* was most common (*n* = 18), found in giblets (44%) and chicken (33%), while *C. jejuni* was found in 11 samples from giblets (28%) and chicken (17%). Assembly sizes ranged from 1,625,208 to 1,934,477 bp (Table 1).

**TABLE 1:** Genomic characterization of 29 *Campylobacter* spp. isolates from retail meat in Puerto Rico^*a*^

SRR number	Strain	Assembly	Assembly length (bp)	N_50_	GC (%)	Contigs	Sequence type (ST)	Total reads	Coverage depth (x)	Estimated completeness (%)	Species	Putative antibiotic resistance genes	Phenotypic antibiotic resistance	BioSample
SRR19139722	22PRCB08-C1	GCA_023279885.1	1,946,785	169145	31	41	12669	3,357,846	411 x	100%	*C. coli*	*bla*_*OXA-193*_	AZI-CLI-ERY-GEN-NAL-TET	SAMN28156920
SRR23384505	22PR12CB09-C1	GCA_028574055.1	1,863,284	163335	31	72	460	165,336	204 x	99%	*C. coli*	*bla*_*OXA-594*_	CLI-FFN	SAMN33227629
SRR23384506	22PR11CB03-C1	GCA_028574135.1	1,671,238	264421	31.5	29	829	4,374,850	383 x	93%	*C. coli*	None^*b*^	CLI	SAMN33227628
SRR23958366	23PR01CH01-C1	GCA_029399555.1	1,752,507	209980	31.5	22	1181	2,994,014	383 x	93%	*C. coli*	*bla*_*OXA-594*_	CLI	SAMN33905782
SRR23958367	23PR01CG01-C1	GCA_029399595.1	1,852,409	120393	31	41	7634	1,536,296	185 x	93%	*C. coli*	*bla*_*OXA-594*_ *, gyrA*	CIP-CLI-FFC	SAMN33905781
SRR28901620	23PR09CL01-C1	GCA_039503805.1	1,653,702	166495	31.5	15	48	444,678	58 x	93%	*C. coli*	*cmeA, cmeB*	CLI	SAMN41218543
SRR28901621	23PR09CG01-C1	GCA_039503885.1	1,695,325	145952	31	48	2517	163,216	38 x	100%	*C. coli*	*cmeB*	CLI	SAMN41218542
SRR28901622	23PR09CH01-C1	GCA_039503925.1	1,696,920	221367	31.5	29	13785	368,556	48 x	92%	*C. coli*	*cmeA, cmeB, tet(O)*	CLI-FFN	SAMN41218541
SRR28901624	23PR08GT04-C1	GCA_039503825.1	1,806,880	153101	31	59	12592	384,062	44 x	100%	*C. coli*	*aph(2")-If, aph(3')-IIIa, gryA, rpIV, tet(O)*	AZI-CIP-CLI-ERY-FFN-GEN-NAL	SAMN41218539
SRR28901625	23PR08CB01-C1	GCA_039503905.1	1,887,993	228741	31	52	1119	222,658	34 x	100%	*C. coli*	*cmeB*	AZI-CIP-CLI-FFN	SAMN41218538
SRR28901629	23PR10CL01-C1	GCA_039503785.1	1,952,160	175744	31.5	14	12856	266,126	34 x	92%	*C. coli*	*cmeA, cmeB*	CIP-CLI-FFN	SAMN41218545
SRR28901631	23PR08CB03-C1	GCA_039503855.1	1,858,853	308385	31	47	1082	355,798	42 x	94%	*C. coli*	*aph(3')-VIIa, cmeB*	CIP-CLI-FFN	SAMN41218535
SRR28901632	23PR05CL01-C1	GCA_039503845.1	1,718,850	225095	31.5	34	13428	330,470	44 x	97%	*C. coli*	*cmeA, cmeB, tet(O)*	CIP-CLI-FFN	SAMN41218534
SRR19139723	22PR03CB03-C1	GCA_023279225.1	1,725,378	177322	31.5	15	7818	355,798	360 x	92%	*C. coli*	*tet(O)*	CIP-CLI-NAL-TET	SAMN28156919
SRR21481168	22PR07CB05-C1	GCA_025117555.1	1,732,682	162758	31.5	27	353	1,787,604	228 x	94%	*C. coli*	*gyrA*	CIP-CLI-FFN	SAMN30723139
SRR25837686	23PR07CG01-C1	GCA_048094505.1	1,822,953	133890	31	94	7818	1,666,194	201 x	94%	*C. coli*	*aph(3')-VIIa, bla*_*OXA-594*_ *, cmeB*	CLI-TET	SAMN37215902
SRR25837710	23PR05CH01-C1	GCA_048090715.1	1,768,575	113520	31	54	51	1,563,702	197 x	90%	*C. coli*	*cmeB*	CLI-FFN-NAL-TET	SAMN37215880
SRR25837716	23PR03CG01-C1	GCA_048090635.1	1,808,623	106601	31.5	38	10452	2,316,400	272 x	100%	*C. coli*	*gyrA*	CIP-CLI-NAL-TET	SAMN37215866
SRR18992203	23PR02CB03-C1	GCA_023204515.1	1,781,454	160578	30	41	460	2,698,514	283 x	99%	*C. jejuni*	*tet(O)*	CLI-TET	SAMN27996795
SRR28901623	23PR09CB02-C1	GCA_048090535.1	1,795,715	128641	30	32	21	385,252	47 x	98%	*C. jejuni*	*cmeA, cmeB, cmeC, gryA*	CIP-CLI-TET	SAMN41218540
SRR28901626	23PR08CL01-C1	GCA_048090575.1	1,802,343	104056	30.5	35	6175	481,050	60 x	99%	*C. jejuni*	*cmeB*	CIP-CLI-TET	SAMN41218537
SRR28901627	23PR08CH01-C1	GCA_048090515.1	1,686,180	150434	30.5	19	1101	315,464	41 x	100%	*C. jejuni*	*cmeA, cmeB, cmeC*	AZI-CLI	SAMN41218536
SRR28901628	23PR11CB02-C1	GCA_048090415.1	1,822,566	166466	30	72	7818	481,050	35 x	95%	*C. jejuni*	*cmeA, cmeB, cmeC, rpIV, tet(O)*	AZI-CIP-CLI-FFN	SAMN41218546
SRR28901630	23PR10CG01-C1	GCA_048090455.1	1,656,948	121368	30.5	26	10452	308,944	39 x	100%	*C. jejuni*	*cmeA, cmeB, gryA, rpIV*	CLI	SAMN41218544
SRR21481169	22PR07CB03-C1	GCA_025117945.1	1,662,023	177892	30.5	33	353	2,590,150	347 x	92%	*C. jejuni*	*rpIV*	CLI-GEN-NAL-TET	SAMN30723138
SRR21481170	22PR04CB07-C1	GCA_025118005.1	1,700,872	141427	30.5	33	3262	3,010,578	356 x	96%	*C. jejuni*	*rpIV*	CLI-FFN-TET	SAMN30723137
SRR23958364	22PR10CB01-C1	GCA_029383795.1	1,806,845	181192	30	35	1119	2,472,182	312 x	100%	*C. jejuni*	*aph(3')-IIIa, rpIV, tet(O)*	AZI-CLI-ERY-GEN-NAL-TET	SAMN33905784
SRR23958365	23PR02CH01-C1	GCA_029399605.1	1,714,300	154394	30.5	17	11193	1,372,884	187 x	98%	*C. jejuni*	*bla*_*OXA-594*_	CLI-TET	SAMN33905783
SRR25837687	23PR07CL01-C1	GCA_031058375.1	1,627,432	151029	30.5	29	3262	1,662,114	224 x	96%	*C. jejuni*	*cmeA, cmeB, cmeC, rpIV*	CIP-CLI-FFN	SAMN37215901

^*a*^ Antimicrobial agent abbreviations: AZI, azithromycin; ERY, erythromycin; FFN, florfenicol; GEN, gentamicin; TET, tetracycline; CIP, ciprofloxacin; Nal, nalidixic acid; CLI, clindamycin.

^*b*^ None, No antimicrobial resistance genes were detected.

**Fig 1:**
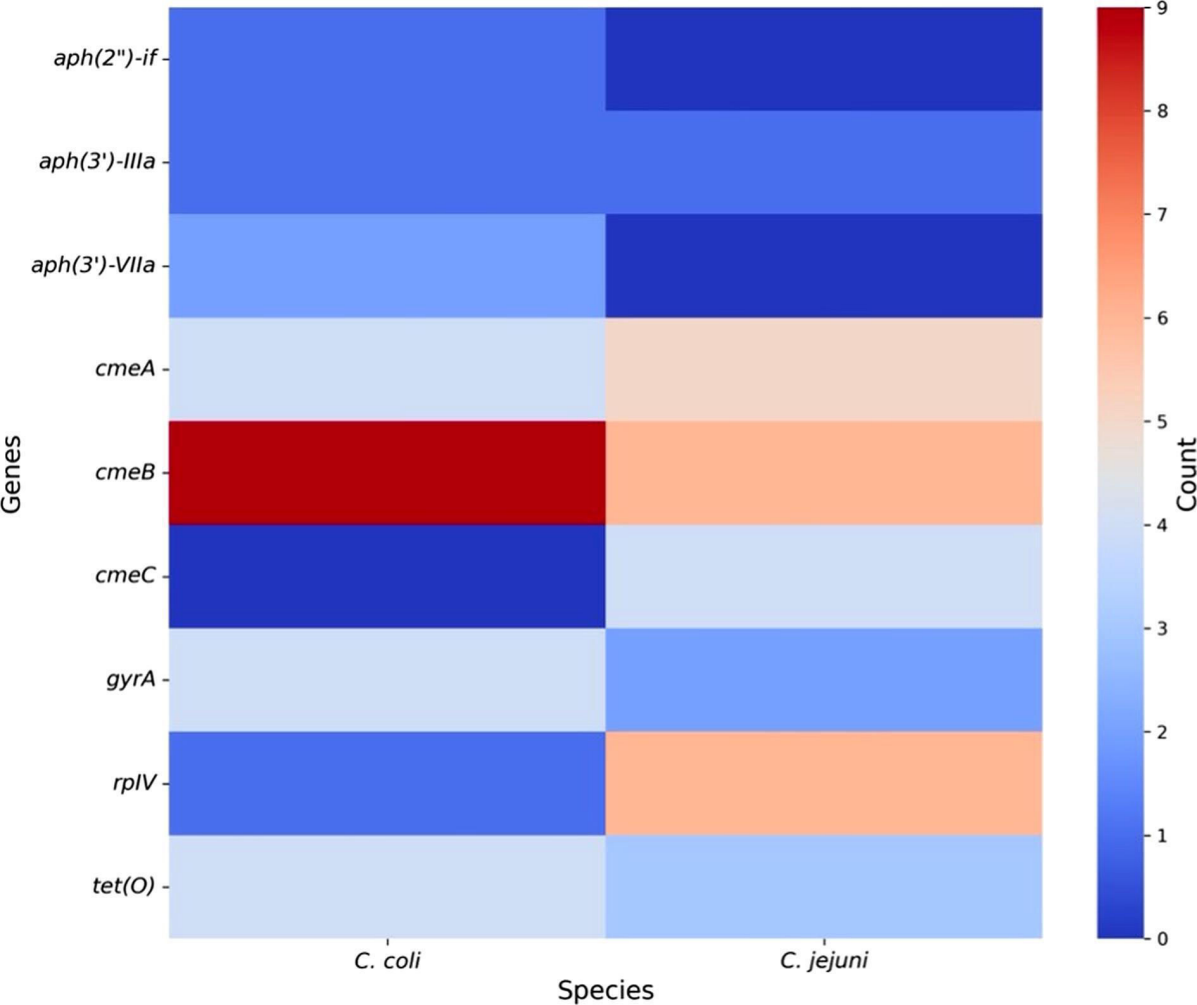
Prevalence of antimicrobial resistance (AMR) genes in *Campylobacter coli* (n = 18) and *Campylobacter jejuni* (n = 11). The heatmap shows the frequency of nine AMR-associated genes across both species. Color intensity reflects gene prevalence, with blue indicating low and red indicating high counts. The heatmap was generated using Python 3.12 with the seaborn and matplotlib libraries.

Our study revealed high AMR rates in *Campylobacter* from retail meats in Puerto Rico, particularly to tetracyclines (TET, 41%), quinolones (CIP, 45%), lincosamides (CLI, 100%), and florfenicol (FFN, 45%). Phenotypic resistance correlated with relevant AMR genes, including *tet(O*) and *gyrA* mutations. This genotypic-phenotypic concordance aligns with Hull et al. (10), who reported over 80% agreement. Our findings also mirror trends observed in a US study; Zhao et al. (11) found 44% TET and 26% CIP resistance in retail meat isolates from 2002 to 2007, and NARMS 2021 (12) reported CIP (64%) and TET (55%) resistance in *C. coli* from chicken. *C. coli* also exhibited higher frequencies of AMR genes such as *aph(3')-IIIa* and the *cmeABC* efflux pump system (Fig. 1), both linked to multidrug resistance (13). Only four isolates carried the *cmeABC* system, crucial to efflux-mediated resistance. These findings highlight the need for ongoing surveillance of AMR in foodborne pathogens across the farm-to-fork continuum, in line with prior baseline studies (14, 15).

## Data Availability

The sequence reads and assembled genomes have been deposited in NCBI under BioProject PRJNA29664. The associated SRA accession numbers are as follows: SRR19139722, SRR23384505, SRR23384506, SRR23958366, SRR23958367, SRR28901620, SRR28901621, SRR28901622, SRR28901624, SRR28901625, SRR28901629, SRR28901631, SRR28901632, SRR19139723, SRR21481168, SRR25837686, SRR25837710, SRR25837716, SRR18992203, SRR28901623, SRR28901626, SRR28901627, SRR28901628, SRR28901630, SRR21481169, SRR21481170, SRR23958364, SRR23958365, SRR25837687.
